# Differential effects on KCC2 expression and spasticity of ALS and traumatic injuries to motoneurons

**DOI:** 10.3389/fncel.2014.00007

**Published:** 2014-01-24

**Authors:** Laura Mòdol, Renzo Mancuso, Albert Alé, Isaac Francos-Quijorna, Xavier Navarro

**Affiliations:** Department of Cell Biology, Physiology, and Immunology, Centro de Investigación Biomédica en Red sobre Enfermedades Neurodegenerativas, Institute of Neurosciences, Universitat Autònoma de BarcelonaBellaterra, Spain

**Keywords:** motoneuron disease, spasticity, hypereflexia, KCC2 transporter, SOD1^G93A^ mice

## Abstract

Amyotrophic lateral sclerosis (ALS) is a neurodegenerative disease manifested by progressive muscle atrophy and paralysis due to the loss of upper and lower motoneurons (MN). Spasticity appears in ALS patients leading to further disabling consequences. Loss of the inhibitory tone induced by downregulation of the potassium chloride cotransporter 2 (KCC2) in MN has been proposed to importantly contribute to the spastic behavior after spinal cord injury (SCI). The aim of the present study was to test whether the alterations in the expression of KCC2 are linked to the appearance of spasticity in the SOD^G93A^ ALS murine model. We compared SOD^G93A^ mice to wild type mice subjected to SCI to mimic the spinal MN disconnection from motor descending pathways, and to sciatic nerve lesion to mimic the loss of MN connectivity to muscle. Electrophysiological results show that loss of motor function is observed at presymptomatic stage (8 weeks) in SOD^G93A^ mice but hyperreflexia and spasticity do not appear until a late stage (16 weeks). However, KCC2 was not downregulated despite MN suffered disconnection both from muscles and upper MNs. Further experiments revealed decreased gephyrin expression, as a general marker of inhibitory systems, accompanied by a reduction in the number of Renshaw interneurons. Moreover, 5-HT fibers were increased in the ventral horn of the lumbar spinal cord at late stage of disease progression in SOD1^G93A^ mice. Taken together, the present results indicate that spasticity appears late in the ALS model, and may be mediated by a decrease in inhibitory interneurons and an increase of 5-HT transmission, while the absence of down-regulation of KCC2 could rather indicate an inability of MNs to respond to insults.

## Introduction

Amyotrophic lateral sclerosis (ALS) is an adult onset neurodegenerative disorder that clinically manifests by progressive muscle atrophy and paralysis (Wijesekera and Leigh, 2009) due to the loss of upper and lower motoneurons (MN). The 90% of ALS cases are sporadic with unknown etiology whereas the remaining 10% are inherited forms, caused by genetic mutations. Among these, mutations in the gene encoding for the enzyme Cu/Zn superoxide dismutase 1 (SOD1) have been reported in about 20% of the patients (Rosen, 1993). Several transgenic animal models of ALS have been developed during the last decades. The most widely used is a transgenic mouse that over-expresses the human mutated form of the *sod1* gene with a glycine to alanine conversion at the 93rd codon (Ripps et al., 1995). This model recapitulates most relevant clinical and histopathological features of both familial and sporadic forms of the human disease (Ripps et al., 1995). Moreover, it has been recently reported that alterations of SOD1 protein are also present in sporadic ALS cases, increasing the interest of this model (Bosco et al., 2010).

Spasticity is a secondary complication of different upper MN syndromes characterized by a velocity-dependent increase in muscle tone resulting from hyperexcitability of the stretch reflex (Lance, 1980). This phenomenon is present in ALS patients and leads to important disabling complications that compromise their manual dexterity and gait (Wijesekera and Leigh, 2009; Kiernan et al., 2011). It has been hypothesized that spasticity may occur due to the loss of upper MN and/or alterations of intraspinal motor circuitry (Schütz, 2005; Chang and Martin, 2009, 2011; Dentel et al., 2013). The appearance of spasticity has also been described in the SOD1 ALS murine model (Dentel et al., 2013). In fact, despite the remaining hindlimb muscle innervation at the end stage of the disease could be enough to allow movement, the animals appear paralyzed due to spastic paresis (Mancuso et al., 2011).

Recent studies have demonstrated the relevance of MN increased excitability for the appearance of spasticity after traumatic injuries to the spinal cord (Lu et al., 2008; Boulenguez et al., 2010; Kakinohana et al., 2012; Bos et al., 2013). Rather than be mediated by an increase in excitatory transmission, these studies postulated that the loss of the inhibitory tone below the lesion is mediated by downregulation of the potassium chloride cotransporter 2 (KCC2) in spinal MNs (Boulenguez et al., 2010; Bos et al., 2013). Modulation of the inhibitory amino acids (GABA and glycine) response is determined by changes in the intracellular chloride concentration [Cl^−^_*i*_]. KCC2 is the main chloride extruder expressed in adult neurons, being responsible for the maintenance of the low [Cl^−^_*i*_] (Ganguly et al., 2001; Rivera et al., 2002; Wang et al., 2002; Payne et al., 2003; Stein et al., 2004; Bray and Mynlieff, 2009). At birth, when GABA and glycine responses are excitatory (Ben-Ari et al., 2007), KCC2 is barely detectable but increases progressively during the early post-natal days of the murine life (Rivera et al., 1999; Wang et al., 2002; Payne et al., 2003). Although excitatory actions of GABA and glycine during early development are relevant for the establishment of circuitry in the spinal cord and the development of motor functional patterns (Stil et al., 2011), the increased excitability induced by KCC2 down regulation in the adult spinal cord after trauma has also been linked to alterations of locomotor pattern, chronic pain and spasticity (Boulenguez et al., 2010; Bos et al., 2013).

In the present experiment, we tested whether changes in the expression of KCC2 in the SOD^G93A^ ALS model are of relevance for the appearance of spasticity at late stages of the disease process. For assessing the potential contributing mechanisms we compared the changes in KCC2 induced by SCI and by peripheral nerve lesions in wild type mice.

## Material and methods

### Transgenic SOD1^G93A^ mice

Transgenic mice with the G93A human SOD1 mutation [B6SJL-Tg(SOD1-G93A)1Gur] were obtained from the Jackson Laboratory (Bar Harbor, ME, USA), and maintained at the Animal Service of the Universidad de Zaragoza. Hemizygotes B6SJL SOD1^G93A^ males were obtained by crossing with B6SJL females from the CBATEG (Bellaterra, Spain). The offspring was identified by PCR amplification of DNA extracted from the tail tissue. All experimental procedures were approved by the Ethics Committee of the Universitat Autònoma de Barcelona, where the animal experiments were performed, and followed the guidelines of the European Commission on Animal Care and the Canadian Council on Animal Care.

### Surgical procedures

Adult (8–10 weeks old) female wild type C57BL/6 mice (Charles River) were anesthetized by i.p. injection of ketamine (10 mg/kg; Imalgene) and xylazine (1 mg/kg; Rompun). For the nerve crush, the sciatic nerve was exposed at the mid-thigh and subjected to a crush during 30 s for three times in succession with a Dumont no. 5 forceps. For SCI a laminectomy at the 11th thoracic vertebra was performed. The exposed spinal cord was contused using the Infinite Horizon Impactor device (Precision Scientific Instrumentation), using a force of 50 kdynes and with tissue displacement ranging between 500 and 700 μm. After injury, the skin was sutured and animals were left to recover on a hot pad and returned to their home cages with free access to food and water.

### Functional assessment

Locomotors recovery was evaluated in an open-field test using the nine-point Basso Mouse Scale (BMS) (Basso et al., 2006; Klopstein et al., 2012), which was specifically developed for locomotors testing after spinal cord contusion injuries in mice. The BMS analysis of hindlimb movements and coordination was performed by two independent researchers and the consensus score was taken. The final score is presented as mean ± s.e.m.

### Nerve conduction tests

The sciatic nerve was stimulated percutaneously by means of single pulses of 0.02 ms duration (Grass S88) delivered through a pair of needle electrodes placed at the sciatic notch. The compound muscle action potential (CMAP, M wave) and the reflex H wave were recorded from the tibial anterior (TA) and the plantar (interpose) muscles with microneedle electrodes (Valero-Cabré and Navarro, 2001; Mancuso et al., 2011). For evaluation of the motor central pathways, motor evoked potentials (MEP) were recorded from the same muscles in response to transcranial electrical stimulation of the motor cortex by single rectangular pulses of 0.1 ms duration, delivered through needle electrodes inserted subcutaneously, the cathode over the skull overlaying the sensorimotor cortex and the anode at the nose (García-Alías et al., 2003; Mancuso et al., 2011). All potentials were amplified and displayed on a digital oscilloscope (Tektronix 450S) at settings appropriate to measure the amplitude from baseline to the maximal negative peak. To ensure reproducibility, the recording needles were placed under microscope to secure the same placement on all animals guided by anatomical landmarks. During the tests, the mice body temperature was kept constant by means of a thermostated heating pad.

### Histology

SOD^G93A^ mice at 8, 12 and 16 weeks of age, and sciatic nerve crushed (at 7 days post injury, dpi) and spinal cord injured (at 28 dpi) wild type mice were included in the histological analysis (4–5 mice per group). Animals were transcardially perused with 4% paraformaldehyde in PBS and the lumbar segment of the spinal cord was harvested, post-fixed overnight, and cryopreserved in 30% sucrose. Transverse 40-μm thick sections were serially cut with a cry tome (Lexica) between L2 and L5 segmental levels. Spinal cord slices were sequentially collected free-floating in Olmos medium.

For immunohistochemistry, sections were blocked with PBS-Triton 0.3%-normal donkey serum 5% and incubated overnight at 4°C with primary antibodies: rabbit anti-KCC2 (KCC2, 1:500, Millipore), mouse anti-neurofilament non-phosphorylated heavy chain (SMI-32, 1:1000, Covance), mouse anti-activating transcription factor 3 (ATF3, 1:500, Abeam), rabbit anti-ionized calcium binding adaptor molecule 1 (Iba1, 1:1000, Wako), rabbit anti-serotonin (5-HT, 1:5000, Sigma) or rabbit anti-calbindin (1:200, Chemicon). After washes, sections were incubated for 1 h at room temperature with Alexi 488 or Alexi 594 conjugated secondary antibody (1:200; Life Science). For co-localization, spinal NMS were labeled with NeuroTrace 500/525 Green Fluorescent Nissl (1:200, Life Science).

To quantify microglial immunoreactivity, microphotographs of the ventral horn gray matter were taken at ×400 and, after defining the threshold for background correction, the integrated density of Iba1 labeling was measured using ImageJ software (Mancuso et al., 2012). The integrated density is the area above the threshold for the mean density minus the background.

### Protein extraction and western blot

For protein extraction, another subset of mice (*n* = 3–4) of the same experimental groups, i.e., SOD^G93A^ mice at 8, 12 or 16 weeks of age, mice with sciatic nerve crush and mice with SCI, were anesthetized and decapitated. The lumbar spinal cord was removed and divided into quarters to isolate the ventral quadrants. In animals that received a sciatic nerve crush, only the ventral horn of the lesioned side was used for protein extraction. Samples were prepared for protein extraction and homogenized in modified RIPA buffer (50 mom Tris–HCl pH 7.5, 1% Triton X-100, 0.5% sodium deoxycholate, 0.2% SDS, 100 mM NaCl, 1 mM EDTA) adding 10 μl/ml of Protease Inhibitor cocktail (Sigma) and PhosphoSTOP phosphatase inhibitor cocktail (Roche). After clearance, protein concentration was measured by Lowry assay (Bio-Rad, Dc protein assay).

Western blots were performed by loading 20 μg of protein of each sample in SDS-poliacrylamide gels. The transfer buffer was 25 mM trizma-base, 192 mM glycine, 20% (v/v) methanol, pH 8.4. The membranes were blocked with 5% BSA in PBS plus 0.1% Tween-20 for 1 h, and then incubated with primary antibodies at 4°C overnight. The primary antibodies used were: mouse anti-GAPDH (1:20000, Millipore), rabbit anti-phospho-^Ser940^ KCC2 (1:1000, Phosphosolutions), rabbit anti-KCC2 (1:500, Millipore), mouse anti-gephyrin (1:1000, BD Bioscience) and anti-GAD65/67 (1:1000, Abcam). Horseradish peroxidase–coupled secondary antibody (1:5000, Vector) incubation was performed for 1 h at room temperature. The membranes were visualized using enhanced chemiluminiscence method and the images were collected and analyzed with a Gene Genome apparatus and Gene Snap and Gene Tools software (Signee), respectively.

### Statistical analysis

Data are expressed as mean ± s.e.m. Electrophysiological test results were statistically analyzed using repeated measurements and One-Way ANOVA, applying Turkey *post-hoc* test when necessary. For immunobloting and histological data we used Mann-Whitney (for two groups comparison) or Kruskal-Wallis tests (for multiple groups comparison) followed by Dunn's *post-hoc* test (Prism 6 software; Graphpad). The level of significance was set at *p* < 0.05.

## Results

### SOD1^G93A^ animals show hyperreflexia and spasticity at late stages of disease progression

**Figure 1:**
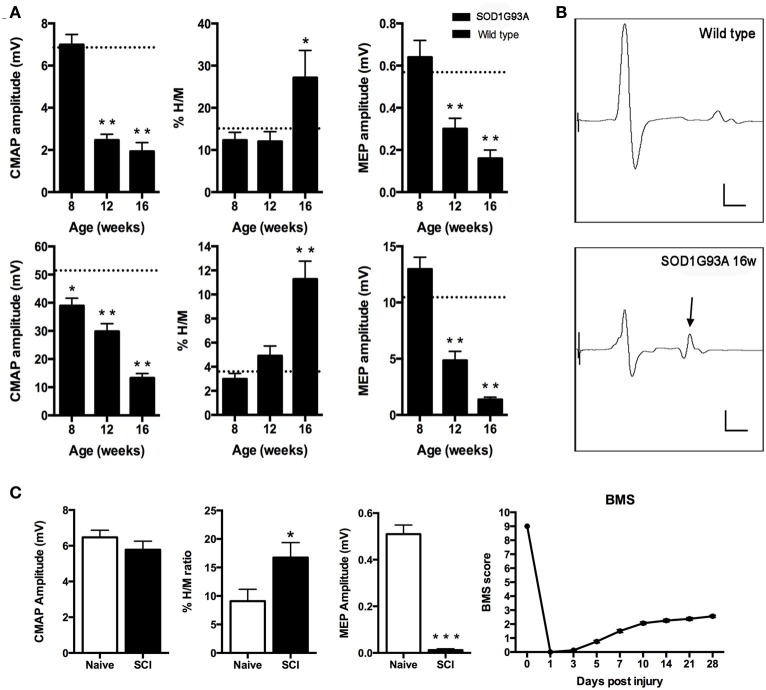
**(A,B)** Peripheral and central motor conduction along disease progression in SOD1^G93A^ mice. **(A)** Plantar (upper) and tibialis anterior (lower) compound muscle action potential (CMAP) amplitude, %H/M ratio and motor evoked potentials (MEP) amplitudes of SOD1^G93A^ animals at 8, 12, and 16 weeks of age. Values are represented as mean ± s.e.m. Dashed line represents wild type mean value for each parameter. ^*^p < 0.05; ^**^p < 0.01 vs. wild type littermates. **(B)** Representative recordings of plantar muscle CMAPs. Note the relative increase of the H wave (arrow) reflecting hyperreflexia. Scale bars: 2 mV; 2 ms. **(C)** Functional assessment of SCI animals. Compound muscle action potential (CMAP) amplitude of the plantar muscle. Hyperreflexia evaluated by means of the %H/M ratio recorded in the plantar muscle after sciatic nerve stimulation. ^*^p < 0.05 vs. naïve mice. The lack of differences indicates that the increased %H/M ratio is not due to CMAP alterations. Motor evoked potentials (MEP) amplitude. BMS score as a measure of the locomotor capacity of the animals in the open field walking test. Note that at 28 dpi the score remains below 3, indicating that animals cannot support their own weight.

We first evaluated peripheral motor nerve conduction to assess the progressive muscle denervation by stimulating the sciatic nerve and recording in TA and plantar muscles (Mancuso et al., 2011). Results revealed a different pattern of muscle denervation in the two tested muscles; the plantar muscle CMAP showed a fast drop in amplitude at 12 weeks of age, whereas the TA CMAP progressively decreased in amplitude from 8 weeks of age (Figure 1A). The monosynaptic spinal reflex activity was assessed by the H/M ratio (Mancuso et al., 2011). Results evidenced a significant increase of the H/M ratio in both tested muscles (*p* < 0.01) at the end stage of the disease (16 weeks), coincident with the spastic condition (Figure 1A). Figure 1B shows representative recordings to illustrate the increased H/M ratio in the plantar muscle of 16 weeks aged SOD1^G93A^ mice. Finally, we assessed central motor conduction by means of MEPs to evaluate the state of the spinal motor descending pathways. Results showed a significant reduction of MEPs amplitude from 12 weeks of age both in TA and plantar muscles (Figure 1A).

### Spinal cord injury causes locomotor impairment and hyperreflexia

We evaluated the locomotor function of wild type mice after SCI by means of the BMS score (Basso et al., 2006; Klopstein et al., 2012). Results revealed that injured mice achieved less than 3 over the 9 points scale at 28 days, evidencing the inability to support their own weight (Figure 1C). Then, we assessed central motor conduction preservation and hyperreflexia of the animals at 28 dpi. Results showed a significant reduction of MEPs and an increased H/M ratio, coincident with the spastic behavior in the animals' hindlimbs (Figure 1C).

### KCC2 expression is not altered in lumbar spinal MNs of SOD1^G93A^ animals

**Figure 2:**
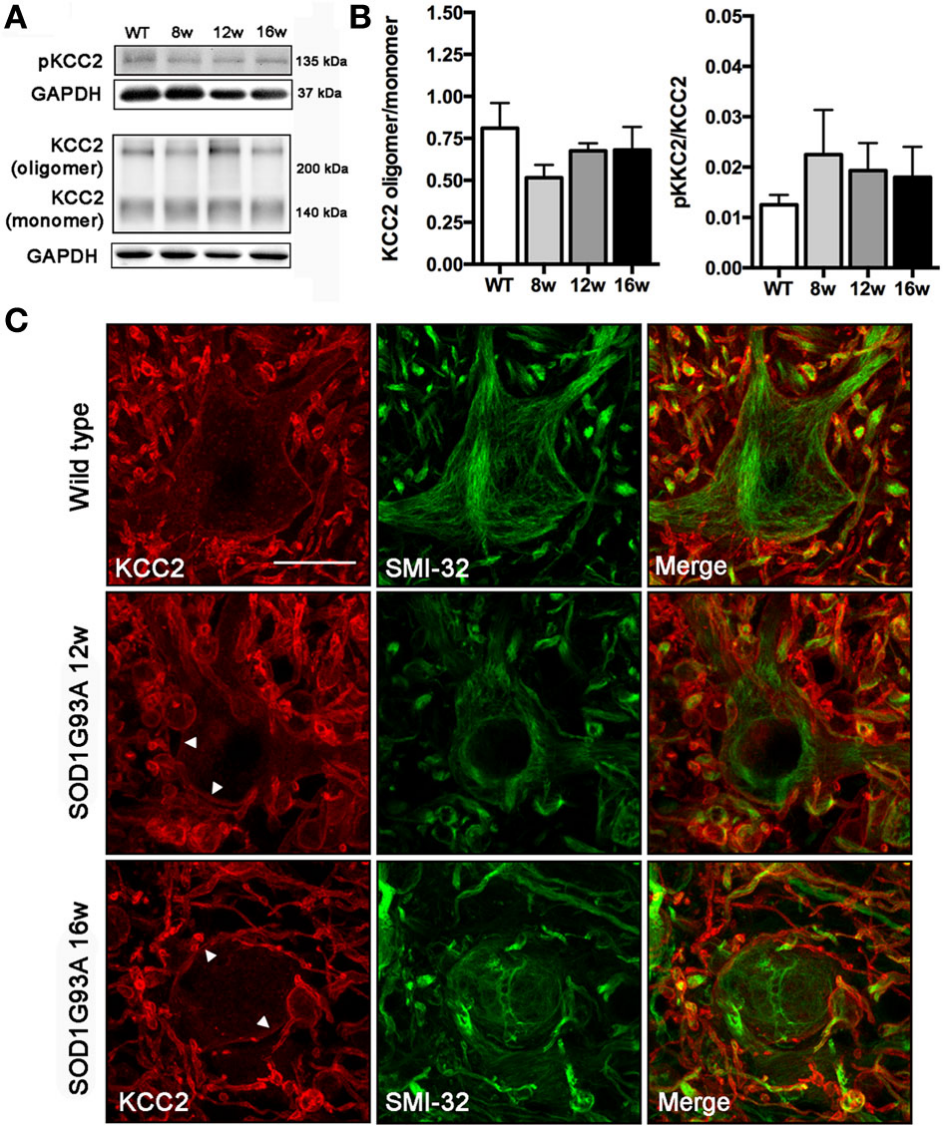
**Analysis of KCC2 and phopho-KCC2 expression in the ventral spinal cord of SOD1^G93A^ mice at 8, 12, and 16 weeks of age. (A)** Representative blots of phopho-KCC2 and total KCC2 (on both monomeric and oligomeric states). **(B)** KCC2 western blots quantifications. Graphs represent the ratio between the oligomeric KCC2 (functional state) vs. monomeric KCC2 and the phopho-KCC2 (active form) vs. monomeric KCC2. Note the lack of significant differences between SOD1^G93A^ at any age and wild type littermates. Data are represented as mean ± s.e.m. **(C)** Confocal images of wild type and SOD1^G93A^ L4 spinal MNs confirmed the maintenance of membrane-bound (active) KCC2 in transgenic animals along disease progression, even in abnormal MNs at 16 weeks of age. Arrowheads show the membrane bound KCC2 in SOD1^G93A^ L4 spinal MNs. Scale bar 20 μm.

**Figure 3:**
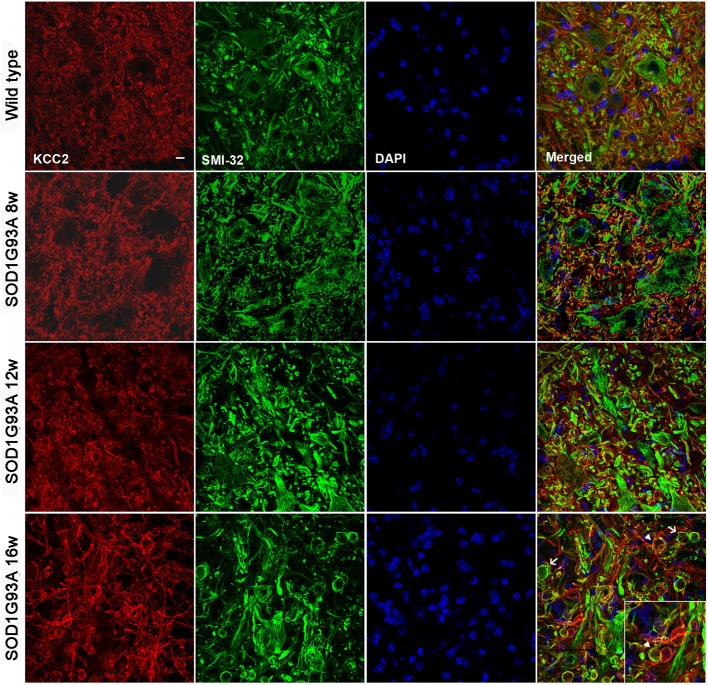
**Confocal images of L4 ventral spinal cord of wild type and SOD1^G93A^ mice at 8, 12, and 16 weeks of age.** Note the progressive increase in number and volume of abnormal swollen structures (arrow) along the disease progression in SOD1^G93A^ animals. The KCC2 remained localized in the cell membrane even in these swallows (arrowheads). Scale bar 10 μm.

We did not observe changes in the KCC2 oligomer/monomer ratio (Figures 2A,B) in SOD1^G93A^ mice at 8, 12, or 16 weeks of age compared to wild type mice. Accordingly, no significant changes in the phosphorylated form were observed in SOD1^G93A^ mice (Figures 2A,B). We then characterized the localization of KCC2 by immunohistochemistry in lumbar MNs of SOD1^G93A^ mice along disease progression. Confocal images showed no changes in subcellular localization of KCC2 at 8, 12 and 16 weeks of age (Figure 2C). In fact, co-labeling of KCC2 and SMI-32 confirmed that KCC2 remained in the MNs plasma membrane at late stages of the disease, as no co-localization of both markers was observed in the samples of SOD1^G93A^ mice, even in degenerating MNs (Figure 3). Taken together, these findings indicate that KCC2 expression and localization in the MN plasma membrane remains unchanged during ALS disease progression.

### Reduced active form of KCC2 in lumbar spinal MNs after SCI

**Figure 4:**
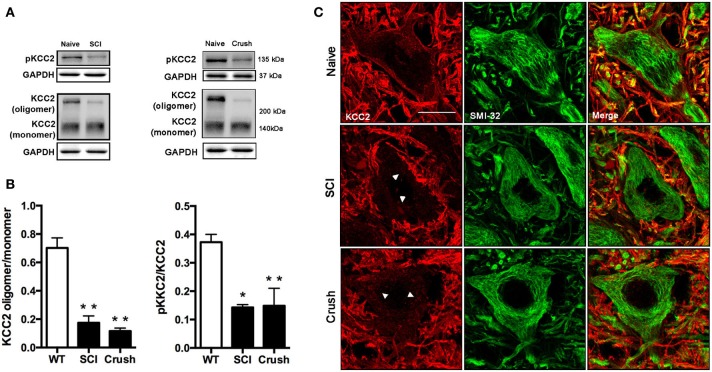
**Analysis of KCC2 and phopho-KCC2 expression in the ventral spinal cord of SCI and sciatic nerve injured mice. (A)** Representative blots of phopho-KCC2 and total KCC2 (on both monomeric and oligomeric states) in SCI and crush mice. **(B)** KCC2 western blots quantifications. Graphs represent the ratio between the oligomeric KCC2 (functional state) vs. monomeric KCC2 and the phopho-KCC2 (active form) vs. monomeric KCC2. Both analyses revealed a decrease in the active form of the KCC2 in SCI (28 days after injury) and in sciatic nerve crush (7 days after injury). Data are represented as mean ± s.e.m.; ^*^p < 0.05, ^**^p < 0.01 vs. naïve animals. **(C)** Confocal images of L4 spinal MNs revealed the presence of membrane-bound (active) KCC2 in naïve but not in SCI and sciatic nerve injured animals. Arrowheads point internalized KCC2 aggregates. Scale bar 20 μm.

We first examined KCC2 expression by western blotting in the lumbar ventral spinal cord of animals with SCI at 28 dpi. Compared to naïve animals, we observed a slight reduction of total KCC2 after SCI (around 20%), although the difference did not reach statistical significance (*p* = 0.06, data not shown). This tendency is in agreement with previously reported results by. Boulenguez et al. (2010) after SCI in the rat. To check the activation state of the KCC2, we also performed western blotting for the phosphorylated form of the KCC2 (pKCC2) with a specific antibody against phopho-Ser^940^. In contrast to total KCC2 expression, the phosphorylated form was decreased after SCI compared to control samples (*p* < 0.05; Figures 4A,B). Since it has been previously described that KCC2 oligomer/monomer ratio is increased at maturity and correlates with KCC2 activation (Blaesse et al., 2006; Boulenguez et al., 2010; Bos et al., 2013), we also analyzed the ratio between the oligomeric and monomeric states of the KCC2. The results showed a significant reduction of the oligomer/monomer ratio (Figures 4A,B; *p* < 0.05), confirming the above results on the reduction of the pKCC2 expression.

Confocal immunohistochemical images confirmed KCC2 staining into the MNs cytoplasm. This labeling pattern contrasted to the uniform band surrounding MNs in naïve animals (Figure 4C). Together, these results indicate that the translocation of KCC2 to the plasma membrane of MN is reduced after SCI.

### Reduced active form of KCC2 is observed in lumbar spinal MNs after sciatic nerve crush

In order to mimic the muscle denervation that occurs early in the ALS progression, we performed a sciatic nerve crush to analyze early changes of KCC2 after MN axotomy. Results of western blotting showed a significant decrease of the KCC2 oligomer/monomer ratio and of the phosphorylated active form of KCC2 in the injured side of the spinal cord in contrast to naïve animals (Figures 4A,B, *p* < 0.01). Immunohistochemical labeling confirmed that after sciatic nerve crush KCC2 was internalized into the MNs cytoplasm in the injured side forming intracellular clusters. In contrast, KCC2 localization in intact animals, showed a well-defined line surrounding MNs indicating a preferably membrane location of KCC2 in non-injured conditions (Figure 4C).

### Absence of down-regulation KCC2 response to axonal insult in ALS MNs

**Figure 5:**
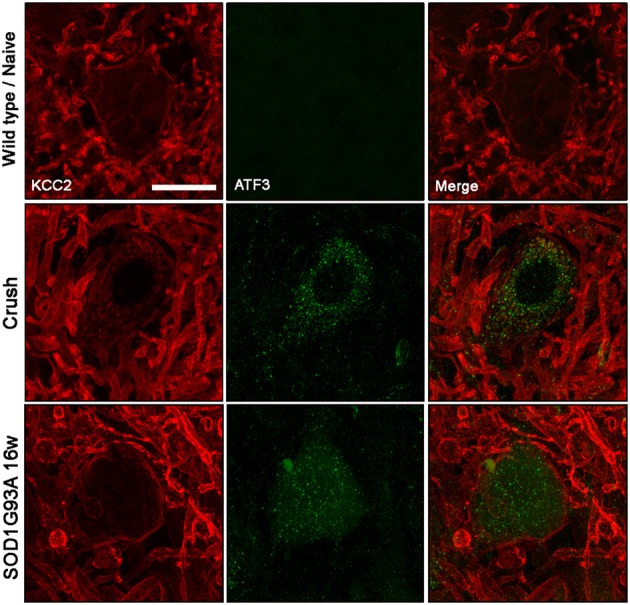
**KCC2 expression and localization in ATF3 labeled MN in wild type/naïve, sciatic nerve injured and 12 weeks old SOD1^G93A^ mice.** Note that membrane-bound KCC2 is reduced in ATF3-positive injured neurons after nerve crush, but it remains in the membrane in ATF3-positive SOD1^G93A^ MNs. Scale bar 20 μm.

We then studied the KCC2 response in injured MNs, identified by labeling the activation transcription factor 3 (ATF3) as a marked of axonal damage (Tsujino et al., 2000), to assess whether KCC2 behave similarly between ALS and axotomized MNs. After sciatic nerve crush, KCC2 cytoplasmic inclusions were found into injured ATF3-positive MNs. On the contrary, the same analysis performed on SOD1^G93A^ ventral spinal cord revealed that KCC2 localization remained normal even if MNs expressed ATF3 (Figure 5). These results suggest an altered response regarding KCC2 expression of ALS MNs after muscle denervation.

### Microglial reactivity is increased in SOD1^G93A^ and after sciatic nerve crush and SCI

**Figure 6:**
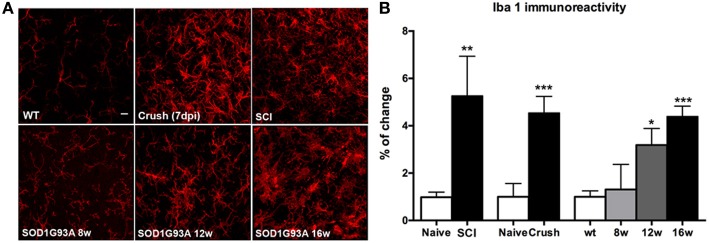
**Comparison of microglial (Iba-1 labeled) immunoreactivity in SOD1^G93A^, SCI and sciatic nerve injured mice. (A)** Representative confocal images of the ventral part of the lumbar spinal cord of wild type/naïve, SOD1^G93A^ at 8, 12, and 16 weeks of age, SCI and sciatic nerve crush injured animals. Scale bar 10 μm. **(B)** Iba-1 immunoreactivity quantification revealed a progressive increase of microglial reactivity during disease progression in SOD1^G93A^ mice. At late stages (16 weeks of age), Iba-1 immunoreactivity level is similar to that observed in SCI and sciatic nerve injured animals. Values are mean ± s.e.m. ^*^p < 0.05, ^**^p < 0.01, ^***^p < 0.001 vs. respective wild type/naïve animals.

It has been reported that microglial cells play a central role in the pathway that leads to KCC2 dephosphorylation (Coull et al., 2005; Ferrini et al., 2013). Thus, we evaluated the microglial activation by immunohistochemistry in SOD1^G93A^, sciatic nerve crush and SCI animals. We focused on the L4-L5 lamina IX in order to analyze the reaction of the microglial cells adjacent to MNs. Results revealed a progressive increase of microglial immunoreactivity in SOD1^G93A^ mice from 8 to 16 weeks of age. On the other hand, SCI and sciatic nerve crush mice also showed an increase in Iba1 reactivity after the lesion at 28 and 7 dpi, respectively. SOD1^G93A^ mice at 16 weeks of age showed similar levels of Iba1 immunoreactivity to those observed in nerve crush and SCI mice, when compared to their respective controls (wild type and naïve) (Figure 6).

### Increased serotonin projections in SOD1^G93A^ lumbar spinal cord

**Figure 7:**
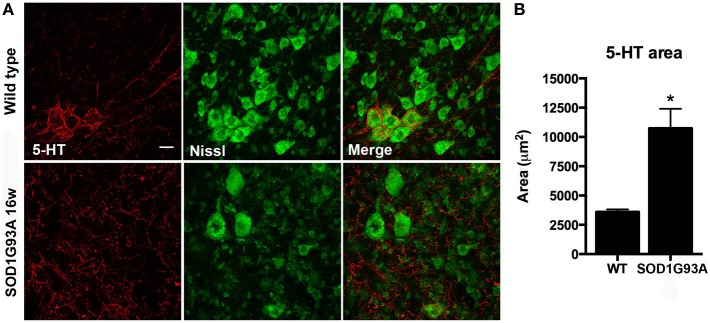
**Serotonin (5-HT) projections to MN pools in the lumbar spinal cord of 16 weeks old SOD1^G93A^ mice and wild type littermates. (A)** Respresentative confocal images show increased 5-HT projections in SOD1^G93A^ compared to wild type mice. Scale bar 20 μm. **(B)** Quantification of the immunolabeling shows a 2 fold increase of 5-HT labeled area in SOD1^G93A^ animals at 16 weeks of age. Values are mean ± s.e.m. ^*^p < 0.05 vs. wild type.

Serotonin (5-hydroxytryptamine, 5-HT) has been postulated as an important factor that contributes to MN excitability by promoting slight depolarization of their membrane potential through increased persistent inward currents (Heckman et al., 2003). 5-HT has been also related to KCC2 phosphorylation and binding to the cell membrane and the consequent decrease in spasticity after SCI (Bos et al., 2013). For this reason, we assessed the 5-HT projections that arrive to L4-L5 spinal MNs. Immunohistochemical analysis revealed an important increase of 5-HT projections in 16 weeks old SOD1^G93A^ lumbar spinal cord (Figure 7, *p* < 0.05).

### Reduced inhibition and renshaw cells degeneration in SOD1^G93A^ lumbar spinal cord

**Figure 8:**
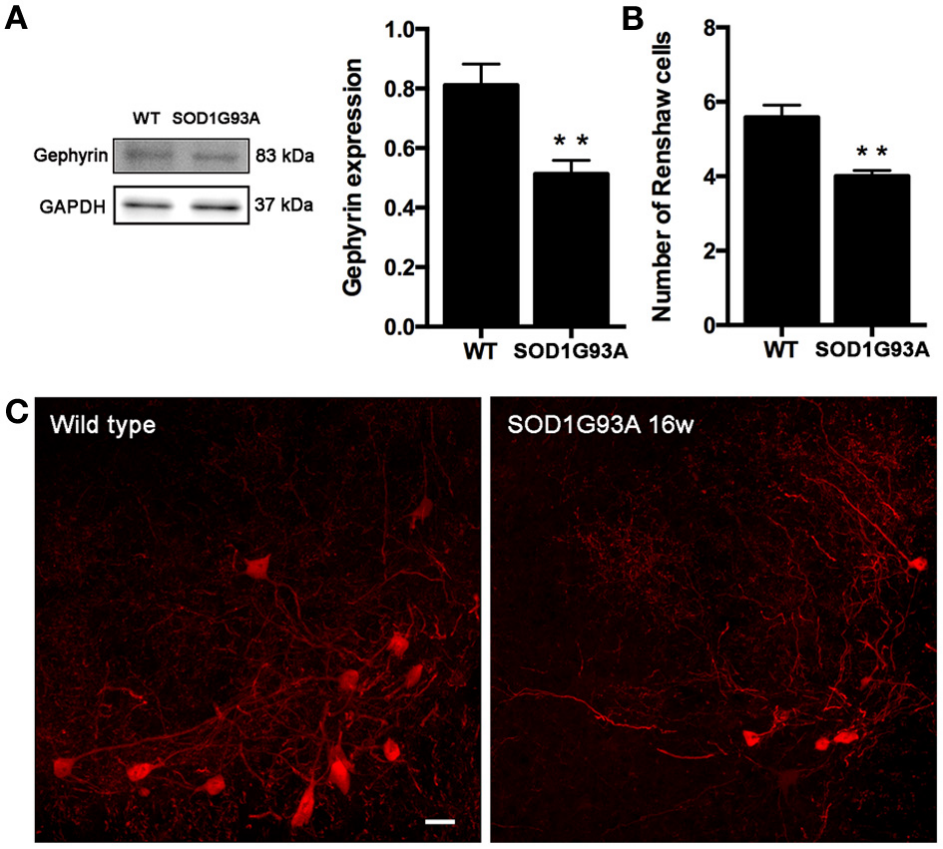
**Evaluation of inhibitory intraspinal circuits in SOD1^G93A^ mice at 16 weeks of age. (A)** Representative blots and quantification of gephyrin expression in the ventral part of the lumbar spinal cord. Results revealed that gephyrin expression in 16 weeks old SOD1 mice was reduced when compared to wild type mice. **(B)** Number of Renshaw cells in the ventral horn of L4 spinal cord of wild type and SOD1^G93A^ animals at 16 weeks of age. Values are mean ± s.e.m. ^**^p < 0.01 vs. wild type animals. **(C)** Representative confocal images of Renshaw cells in the L4 spinal cord of wild type and 16 weeks old SOD1^G93A^ animals. Scale bar 10 μm.

Once revealed that KCC2 expression and localization is not altered along ALS progression, we studied the inhibitory circuits in the lumbar spinal cord of 16 weeks aged SOD1 animals to evaluate its potential involvement on hyperreflexia. We analyzed gephyrin as a general marker of inhibitory glycine and GABA systems. Results showed a significant decrease of gephyrin expression in SOD1^G93A^ mice compared to non-transgenic littermates (Figures 8A,B). We also labeled the Renshaw cells in lamina VII of the L4 spinal cord, since these cells are glycinergic and importantly contribute to the inhibition of MNs. As described previously (Chang et al., 2009), we found a significant decrease in the number of calbindin positive cells in 16 weeks aged SOD1^G93A^ when compared to WT littermates (Figure 8C, *p* < 0.05). The findings suggest that inhibition is reduced in SOD1^G93A^ lumbar spinal cord due to alterations of the glycinergic system.

## Discussion

The results of the present work demonstrate that KCC2 is downregulated after peripheral and central nerve injuries. However, although KCC2 downregulation has been demonstrated to be a key factor in the appearance of hyperreflexia and spasticity after such injuries, we did not found changes in the KCC2 dephosphorylation that could explain the appearance of spastic behavior at the late stage (16 weeks of age) of the ALS murine model. On the other hand, our results suggest that the increased spinal excitability and the appearance of spasticity in ALS may be a consequence of two abnormalities: the loss of inhibitory tone due to loss of Renshaw glycinergic interneurons, and the increased 5-HT projections present in the ventral horn that would directly contribute to increasing MN excitability.

### Factors contributing to spasticity in SOD1^G93A^ mice

Spasticity is present in ALS patients and leads to disabling complications in hand function and gait (Wijesekera and Leigh, 2009; Kiernan et al., 2011). Several works have investigated the mechanisms underlying spasticity after traumatic SCI. A relevant finding of these studies is that KCC2 loss of function is an important hallmark of MN increased excitability and thus, of spasticity after SCI (Boulenguez et al., 2010; Bos et al., 2013). The KCC2 is a potassium-chloride cotransporter, responsible for the low intracellular chloride concentration that allows GABA and glycine inhibitory synaptic responses in the adulthood (Rivera et al., 1999; Ganguly et al., 2001; Wang et al., 2002; Payne et al., 2003; Stein et al., 2004; Bray and Mynlieff, 2009). Phosphorylation of S940 in the intracellular C-terminal domain of the KCC2 has been demonstrated to be responsible for the stabilization of KCC2 on the neuronal cell surface, increasing its functional expression (Li et al., 2007; Lee et al., 2007, 2010). The expression and function of KCC2 is reduced after neural injuries, participating in the lowered strength of inhibitory transmission (Coull et al., 2003). The most prevalent mechanism underlying KCC2 regulation has been postulated to be mediated by brain-derived neurotrophic factor (BDNF) released through microglial signaling (Ulmann et al., 2008; Ferrini and De Koninck, 2013). Indeed, microglia react to alterations of the extracellular milieu with a protective and defensive role secreting specific messengers (including BDNF), that in turn sculpt neuronal circuit excitability (Ferrini and De Koninck, 2013). Although, this mechanism has been commonly described in the dorsal horn of the spinal cord, other studies also described the microglia-BDNF-TrkB-KCC2 signaling in the spinal motor system (Ferrini et al., 2013). In agreement, we found that the decrease of KCC2 phosphorylation after central or peripheral nerve injuries in the ventral horn of the spinal cord was also accompanied by increased microglial reactivity. Although this phenomenon has been reported to contribute to spasticity after SCI (Boulenguez et al., 2010; Bos et al., 2013), here we demonstrate that KCC2 is not altered in SOD1^G93A^ mice MN, and unlikely contributing to the hyperreflexia and spasticity that occurs in SOD1^G93A^ animals. Our results revealed that, opposite to what occurs after SCI, KCC2 remained localized in the cell membrane of MN, even in animals of 16 weeks of age when hyperreflexia and spasticity are clearly present. Fuchs et al. (2010) previously reported a down regulation of KCC2 mRNA in spinal MNs of SOD1^G93A^ mice. They found a slight reduction of mRNA signal in only a few large MNs of 80 days old animals (11 weeks of age), despite that almost 50% of lumbar MNs are not functionally connected to muscle at this time (as evidenced by the CMAP reduction). When they analyzed KCC2 mRNA at 120 days (17 weeks of age) they found a significant reduction of mRNA signal area per neuron, although some of the MNs could be in a degenerative state. KCC2 immunoreactivity was reduced in the neuropil surrounding MNs, similar to what we observed (see Figure 3), whereas some MNs bodies had increased KCC2 labeling in the cytoplasm (Fuchs et al., 2010). These findings suggest that KCC2 disregulation is slight and does not occur until very advanced stages in SOD1^G93A^ mice, quite later than the muscle denervation process.

Mechanisms underlying spasticity have been mostly studied in experimental models of SCI. It is considered that SCI associated spasticity arises from several mechanisms, one of the most important being alterations of 5-HT inputs to spinal MNs. 5-HT descending axons from brainstem nuclei densely innervate spinal MNs, maintaining their excitability through increased persistent calcium current (Heckman et al., 2003). Although damage of serotonergic axons caused by a SCI leads to a transient hypoexcitability of spinal MNs, after a few weeks, MNs compensate for the loss of serotonin inputs through the overexpression of 5-HT receptors. These synaptic modifications promote hyperexcitability and consequent spasticity (Murray et al., 2010, 2011). In accordance, a recent study showed that the heterogeneity of 5-HT receptors is an important feature of hyperexcitability in MNs after SCI. Bos et al. (2013) reported that activation of 5-HT2B and 5-HT2C receptors induced a depolarizing shift in MNs. However, activation of 5-HT2A participated in the activation and restoration of KCC2 expression. Our results reveal that MNs of SOD1^G93A^ mice receive an increased amount of 5-HT projections at 16 weeks of age. Moreover, in contrast to the localized presence of 5-HT projections around MNs in WT animals, in SOD1^G93A^ mice they became spread over lamina IX in the ventral horn of the spinal cord. As a result, 5-HT labeling was found increased and could partially explain the hyperreflexia and spasticity observed at the late stage of the disease, but also the maintenance of KCC2 in the MN membrane. Further studies assessing the differential expression of 5-HT receptors in SOD1^G93A^ mice would be of interest for understanding their interaction with the KCC2 role in ALS.

We further assessed the inhibitory state of the lumbar spinal cord by measuring gephyrin expression. Gephyrin is a structural component of the postsynaptic protein network of both glycine and GABA inhibitory synapses in the spinal cord (Bohlhalter et al., 1994). Our results show a significant reduction of gephyrin expression in 16 weeks old SOD1^G93A^ animals, evidencing decreased inhibition in the ventral spinal cord. Since gephyrin participates in glycine receptor clustering but not in GABAergic synapses formation (Lévi et al., 2004), we further investigated the glycinergic Renshaw cells at late stages in SOD1 mice. Renshaw cells are spinal interneurons located in the ventral horn gray matter that mediate recurrent inhibition to spinal MNs (Katz and Pierrot-Deseilligny, 1999; Alvarez and Fyffe, 2007). These cells have been related to spasticity (Mazzocchio and Rossi, 1997) and, recently, postulated as contributors to spasticity in ALS (Mazzocchio and Rossi, 2010). In fact, our results revealed a significant decrease in the number of calbindin labeled Renshaw cells in the lumbar spinal cord of SOD1 mice at 16 weeks of age. The loss of inhibitory interneurons could partially explain the abnormally increased spinal excitability found in SOD1^G93A^ animals at late stages. Indeed, Chang and Martin have reported an early presymptomatic loss of glycinergic synaptic buttons onto MNs (Chang and Martin, 2009) and the abnormal properties of glycinergic channels in dissociated G93A MNs (Chang and Martin, 2011).

Taken together, our results suggest two distinct mechanisms that may be contributing to hyperreflexia and increased spinal excitability in the SOD1 mouse model. On the one hand, increased lumbar 5-HT may enhance MN excitability through the activation of 5-HT2B and 5-HT2C receptors and, on the other hand, the loss of Renshaw cell leads to a reduction in inhibitory inputs onto MNs. Both phenomena may explain the disabling spastic paresis observed in SOD1^G93A^ animals at the late stage. These exogenous influences may add to the mild MN depolarization state described in this ALS murine model (Boërio et al., 2010).

## Differential MN response between SOD1^G93A^ and nerve crushed MNs

Once demonstrated the lack of KCC2 downregulation in SOD1^G93A^ MNs when compared to SCI, we explored whether the KCC2 response could be an intrinsic feature of ALS MNs. To test this hypothesis, we compared the differences in KCC2 expression between ALS and axotomized wild type MNs. Our results demonstrate for the first time that KCC2 down regulation also occurs in spinal MNs after peripheral nerve injury, as showed by the decrease in the active pKCC2 after sciatic nerve crush. Immunohistochemical evaluation also revealed that MNs labeled for ATF3, a typical marker of axonal injury (Tsujino et al., 2000; Navarro et al., 2007), presented an evident translocation of KCC2 from the plasma membrane to cytoplasmic aggregates. This fact indicates that injured MNs were actively down regulating KCC2 from their cell membrane. After lesion of peripheral axons, differential regulation of protein expression occurs and plays a role in transitioning the neuron from a transmission mode to a regenerative, growth mode (Fu and Gordon, 1997; Pieraut et al., 2007, 2011). One example of the plastic changes that occur after axotomy, are the excitatory responses to GABA and glycine induced by the loss of KCC2 in the neuronal cell membrane. This shift in the balance between excitatory and inhibitory influences that renders injured networks hyperexcitable has been implicated in the pathogenesis of neuropathic pain in dorsal horn neurons (Coull et al., 2003; Cramer et al., 2008; Hasbargen et al., 2010; Janssen et al., 2011, 2012).

Our results in SOD1^G93A^ mice revealed that KCC2 remained in its active phosphorylated form and located in the cell membrane even in MNs that highly expressed ATF3, evidencing a recent process of target muscle disconnection (Vlug et al., 2005; Saxena et al., 2009). After axotomy, KCC2 loss of function would contribute to an increase of spinal synaptic excitability and hyperreflexia (Valero-Cabré and Navarro, 2001), as a feature of the plastic changes that may play a role on nerve regeneration and functional recovery. The lack of such changes in ALS MNs could suggest an inability of these cells to initiate some cellular events in response to muscle disconnection that would allow for axonal regeneration and re-establishment of new neuromuscular junctions. The abnormal response to insults would be also manifested by a progressive increase of neurofilament aggregates (Julien, 1997) surrounded by active and phosphorylated KCC2 with age in SOD1^G93A^ MNs. This observation could explain the fact that we did not observe any changes in the KCC2 activity by analyzing the WB result when comparing with wild type animals. Nevertheless, despite the active KCC2 downregulation could be likely due to muscle disconnection *per se*, it cannot be discarded that such changes are produced by transynaptic effects of the injured primary sensory afferents, explaining the differences we observed between ALS and nerve crushed mice. Thus, further experiments are needed to understand which mechanisms underline the reduction of KCC2 activity.

In summary, the present results demonstrate that there is not downregulation of KCC2 expression from the plasma membrane of ALS MNs, even at advanced stage of the disease, when they have suffered deafferentation from upper MNs and axonal damage and muscle disconnection. This is in contrast to what we found after either SCI or peripheral nerve injury, which induced a rapid decrease of KCC2 phosphorylation in MNs. Such a KCC2 change has been previously described in spinal MNs linked to the development of spasticity (Boulenguez et al., 2010), and in dorsal horn neurons related to the appearance of neuropathic pain (Janssen et al., 2012). The fact that KCC2 is not downregulated along the lifespan of SOD1 mice could indicate that ALS MNs do not react as axotomized normal MNs after muscle denervation.

### Conflict of interest statement

The authors declare that the research was conducted in the absence of any commercial or financial relationships that could be construed as a potential conflict of interest.
